# The Impact of Diversified Farming Practices on Terrestrial Biodiversity Outcomes and Agricultural Yield Worldwide: A Systematic Review Protocol

**DOI:** 10.3390/mps4010008

**Published:** 2021-01-14

**Authors:** Andrea C. Sánchez, Natalia Estrada-Carmona, Stella D. Juventia, Sarah K. Jones

**Affiliations:** 1 Bioversity International, Parc Scientifique d’Agropolis II, 34397 Montpellier, France; andrea.sanchez@cgiar.org (A.C.S.); n.e.carmona@cgiar.org (N.E.-C.); juventia.stella@wur.nl (S.D.J.); 2 Farming Systems Ecology Group, Wageningen University & Research, 6700 AK Wageningen, The Netherlands

**Keywords:** agroecology, management practices, functional groups, biodiversity metrics, monoculture, natural habitats

## Abstract

The expansion and intensification of agriculture have led to global declines in biodiversity. This paper presents a systematic review protocol to clarify under what management and landscape contexts diversified farming practices are effective at improving outcomes for terrestrial biodiversity, and potential trade-offs or synergies with agricultural yields. The systematic review will be developed following the Reporting Standards for Systematic Evidence Syntheses (ROSES). The review will include articles that compare levels of diversity (e.g., abundance, richness, Shannon’s diversity index) of any terrestrial taxon (e.g., arthropods, mammals) in diversified farming systems to levels in simplified farming systems and/or natural habitats, prioritising articles that also report agricultural yields. We will search for relevant peer-reviewed primary studies in two global repositories: Scopus and Web of Science, and among primary studies included in previous meta-analyses that are retrieved from the search. Full-texts of identified articles will be screened using a clear inclusion/exclusion eligibility criteria. All included articles will be assessed to determine their internal validity. A narrative synthesis will be performed to summarize, describe and present the results, and where the articles provide sufficient and appropriate data, we will conduct a quantitative meta-analysis.

## 1. Introduction

Agriculture dominates approximately 40% of the world’s terrestrial surface [1]. The expansion of modern agriculture has caused the widespread conversion of diversified natural ecosystems into simplified monocultures with a small number and variety of cultivated plants [2]. These simplified systems are often highly dependent on external inputs and management practices, such as use of fertilizers and pesticides, irrigation, and tillage [3]. Since the last century, the expansion and intensification of agriculture has led to an increase of 23% in food production [4], while at the same time becoming one of the most important drivers of biodiversity loss worldwide [5,6]. With continued rapid population growth, global food demands are projected to double by 2050 [7], increasing the negative impacts on biodiversity [8]. Alternative farming practices are needed in order to promote livelihoods, food security and biodiversity conservation.

On-farm diversification practices have been proposed as suitable strategies to reduce the negative impact of modern agriculture on biodiversity while promoting sustainable food production [9,10,11,12]. Diversified farming systems involve the association of different varieties and/or species of plants, at multiple temporal and/or spatial scales, or integrating livestock and fish production with crop production [13,14]. Temporally diversified systems—such as systems under crop rotation—are recognised for increasing soil biodiversity responsible for nutrient recycling [15,16]. Plant diversification within and between agricultural plots—such as intercropping, agroforestry, hedgerows, and set-aside—can provide shelter, nesting sites and alternative food sources that promote the diversity of pollinators and natural enemies of pests [17,18,19]. Furthermore, at the landscape scale, the maintenance of natural and semi-natural habitats around and between agricultural fields positively influences the diversity of beneficial species [18,20,21,22]. Evidence is building that diversified farming can also have economic benefits by reducing input requirements and increasing or stabilising yields [14,23,24,25].

Previous reviews have synthesized the effects of diversified farming practices on biodiversity outcome metrics [21,26,27,28,29,30,31,32]. Others have summarised the effects on crop yields or yield stability [14,23,24,25,33,34,35,36]. Most of these reviews suggest that higher farm diversity has positive outcomes for biodiversity, yet that outcomes vary with several factors such as crop type, soil and agrochemical management, outcome metric (e.g., abundance, richness) and which taxa are assessed. The reviews also indicate that more diversified farms have more stable, and often higher, yields, but effects vary by crop, management, and agroecosystem context. While previous studies have greatly advanced our understanding, a new global meta-analysis of effects of diversified farming on biodiversity is valuable for several reasons. First, most of the syntheses focus on only a few taxonomic groups (e.g., arthropoda) [21,30,31] or broad taxonomic groups (e.g., vertebrate, invertebrate) [37]. More granular results are useful for conservation purposes, since diversity needs conserving at varietal, species and community levels. Second, few meta-analyses separate crop diversity from agrochemical or soil management effects, making it difficult to know whether domesticated plant diversity or management factors are responsible for positive outcomes on biodiversity [26,29,31,37,38]. Third, few meta-analyses compare diversified farming against natural habitats to help understand the potential contribution (and shortfall) of farm diversity in conserving biodiversity at levels found in natural habitat. Finally, there is a lack of clarity on when diversified farming can have positive effects on both biodiversity and crop yields to support both biodiversity conservation and food production goals. This is a gap that urgently needs closing to identify trade-offs and synergies and design appropriate incentives that enable farmers to rapidly transition towards more sustainable and biodiversity-friendly agriculture.

### Aim

The main objective of this systematic search is to provide clearer messages on when diversified farming practices are effective at improving outcomes for terrestrial biodiversity in specific agroecological contexts, while minimising trade-offs with agricultural yields. Publishing protocols for scientific reviews is a requirement of the Reporting Standards for Systematic Evidence Syntheses (ROSES) [39], yet rarely completed outside medical research. This systematic review protocol contributes to ensuring best standards for systematic reviews are followed in other fields, notably agronomy and ecology.

## 2. Protocol Design

**Figure 1:**
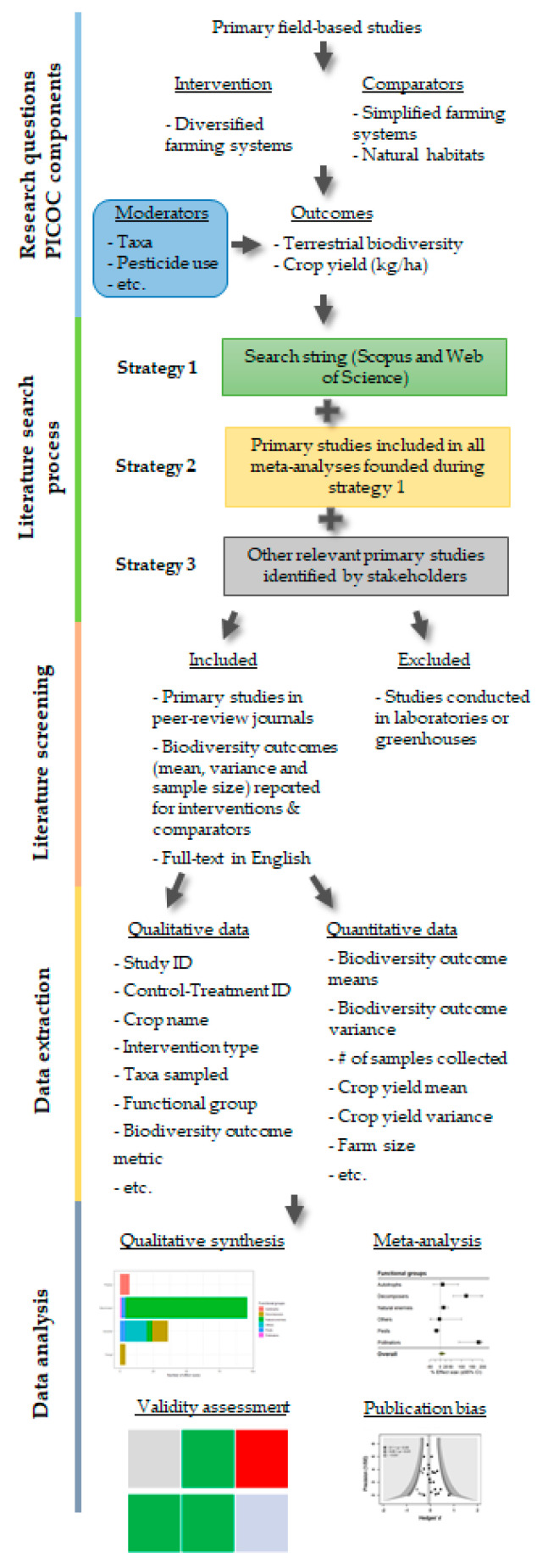
Systematic review protocol methodological steps following Reporting Standards for Systematic Evidence Syntheses (ROSES) guidelines.

The review protocol is a methodological framework for synthesizing evidence presented in primary studies. Such procedures aim to ensure the replicability, transparency and objectivity of searching, inclusion and synthesis of evidence in assessing specific research questions [40]. The systematic review will be developed following the ROSES guidelines [39] (see Figure 1). The completed ROSES’ checklist form is available in Appendix A.

## 3. Stakeholder Involvement

The review will be conducted as part of the CGIAR Water, Land and Ecosystems funded Sustainable foods through diversity-based practices (Sustainable Foods) project. Sustainable Foods will consult with biodiversity and food system experts including from Bioversity International (now part of the Alliance of Bioversity International and the International Centre for Tropical Agriculture), the United Nations Sustainable Development Solutions Network, Natural History Museum (UK) and Centre de coopération internationale en recherche agronomique pour le développement (France) to seek methodological guidance at key points in the review. For example, 10 stakeholders from across these institutions were convened in a workshop on 3–4 October 2019 to provide feedback and improve the research questions, scope, search strategy, database format, validation process and provisional analysis strategy. These experts will be invited to review and contribute to the peer-reviewed papers presenting the final methods and results.

## 4. Step 1: Research Questions

### 4.1. Primary Question

How effective is diversified farming at improving outcomes for terrestrial biodiversity and what are the trade-offs or synergies with yield?

#### Components of the Primary Question

The PICOC (Population, Intervention, Comparator, Outcomes, Context) framework [41] for the primary study question are detailed as follows:

- Population: terrestrial biodiversity in diversified and simplified farming systems and/or natural habitats worldwide, such as bacteria, animals, fungi, plants, etc.;
- Intervention: diversified farming practices (e.g., agroforestry, crop rotation, intercropping);
- Comparator: simplified farming practices (e.g., monoculture), and/or natural habitats (e.g., natural grasslands, primary or secondary forest);
- Outcomes: biodiversity outcome metrics (e.g., abundance, species richness, Shannon’s diversity index, percentage fungi colonization, etc.) and crop yield (i.e., kg/ha);
- Context: primary field-based studies with experimental and observational designs conducted anywhere in the world in agricultural fields, with or without comparable studies conducted in natural habitats.

### 4.2. Secondary Questions

1. What is the state of evidence of diversified farming practices on biodiversity outcomes and trade-offs or synergies with yield, in terms of number of studies, geographic coverage, intervention types, population types (taxa group, functional group), outcome metrics, crop types, intervention practices?
2. Do the effects of diversified farming on biodiversity outcomes and crop yield vary across different:
  - taxonomic groups (e.g., bacteria, fungi, mammals),
  - functional groups (e.g., pest, pollinators, decomposers),
  - outcome metrics (e.g., abundance, species richness, Shannon’s diversity index),
  - crop type (e.g., nuts, vegetables, fruits, etc.),
  - management practices (i.e., fertilizer application, pesticide application, soil management),
  - diversified farming practice (e.g., agroforestry, crop rotation, intercropping).

## 5. Step 2: Searching for Relevant Articles

The search strategy aims to identify a wide number of relevant articles published in peer-reviewed journals. We will not include studies reported in grey literature. The article’s search will be accomplished in three different stages. First, we will search in Scopus (https://www.scopus.com/) and Web of Science (https://apps.webofknowledge.com/), using search strings to identify potentially relevant articles, restricting the search to titles, abstract and keywords in English language articles (Table 1). The search string has been developed in consultation with scientists working on diversified farming practices and/or biodiversity outcomes, convened in October 2019 through the CGIAR-funded Sustainable Foods project. The string has been constrained to search for articles that explicitly consider gradients of farm or landscape diversity, and trade-offs or synergies with yield, to limit the number of non-relevant articles retrieved (see Appendix B). Secondly, we will expand the search of articles by extracting the reference list from all meta-analyses found during the preliminary searches. Third, we will include relevant primary studies known to scientists consulted through the Sustainable Foods project and which are not picked up by the search string. The last two supplementary search processes will help us to identify and include all relevant primary studies including those included in previous related meta-analyses to build on existing knowledge. 

We will assess the comprehensiveness of our search by comparing the number of articles retrieved against the articles retrieved and included in previous, similar reviews. We will compile the CSV export of all the document results from all search stages, and remove any duplicates based on the DOI and article title. All remaining articles will be assigned a unique ID (Study_ID). The search will be updated if it was performed more than 12 months prior to submission of the final results to a peer-reviewed journal.

## 6. Step 3: Selection of Articles

### 6.1. Screening Process

Portable document format (PDF) versions of all accessible articles retrieved during the search stage will be downloaded and renamed with the corresponding Study_ID. Authors are affiliated with Bioversity International, Imperial College London, Wageningen University and Research, and King’s College London. We will download only articles that are open access or accessible through subscriptions made by these institutions. The articles will be screened for inclusion at full-text level against our eligibility criteria by two independent reviewers. The articles will be classified as: (1) include, (2) exclude, and (3) maybe. The decision to include the articles classified as “maybe” will be taken in consensus with a third reviewer based on the eligibility criteria. All excluded articles will be coded with an exclusion reason.

### 6.2. Eligibility Criteria

Only articles published in peer-reviewed journals with full-text in English will be included in this review. Where articles report secondary data (i.e., data from another study), we will exclude the article. The following inclusion criteria will be applied, described using the five PICOC components:

- Eligible Populations: any non-domesticated terrestrial micro or macro-organisms. This includes any surveyed organisms or group of organisms that can be classified to taxonomic phylum level, order or below (i.e., family, species level). Any life stage will be considered.
- Eligible Interventions: the articles must provide a clear description of the diversified agricultural systems. Diversified farming systems are represented by the association of different plant species (e.g., two crops; a crop and a beneficial non-crop), or different varieties/cultivars/accessions of crops (e.g., two crop genotypes) at multiple temporal and/or spatial scales, or the integration of livestock and fish production with crop production. We will distinguish six types of diversified farming systems as described in Table 2.
- Eligible Comparators: articles must provide enough information to differentiate the intervention systems to the comparators. We will classify the simplified farming systems assessed into monoculture and simplified others, and natural habitats into two types of habitats natural habitats, and abandoned farmland (see Table 2). We will not include outcomes from tree plantations for timber or any other commercial purposes.
- Eligible Outcomes: articles must report a quantitative assessment of the effect of interventions and comparators on outcomes for non-domesticated terrestrial biodiversity. Outcomes may include metrics such as: abundance, species richness, activity-density, Chao1 index, colonization percent, Fisher alpha, Jaccard similarity index, Jack-knife species richness, Margalef Index, number of orders, Pielou index, rarefied species richness, Shannon–Wiener index, Shannon evenness index, Shannon index, Simpson index, Simpson’s reciprocal index, Simpson index, species evenness, reproductive success. Included articles must report location data (e.g., geographic coordinates, country), outcome means (or medians), sample sizes, and variance measures (e.g., standard deviation, standard error, interquartile ranges, confidence intervals) for interventions and comparators assessed. The biodiversity outcomes at intervention and control sites must be comparable, i.e., data collected at the same or very similar points in time, using the same or very similar sampling methods.
- Context: we will include only data from primary field-based studies with experimental and observational designs conducted in natural habitats, agricultural fields on farms or outdoor experimental research sites. We will exclude primary studies carried out in laboratories or greenhouses.

The eligibility criteria may be adapted during the articles screening process to overcome any shortcomings that emerge, e.g., to include different biodiversity outcomes, variance measures; and/or other intervention or control systems.

## 7. Step 4: Data Extraction and Coding Strategy

Qualitative and quantitative data will be extracted from all articles that fulfil all the inclusion criteria. The extracted data will be recorded in a contiguous database specially designed and coded for this review (see Appendix C). Intervention and comparator observations from the same study will be recorded and coded in different individual rows, linked by study (Study_ID) and experiment (Control-Intervention_ID) unique identifiers. This database structure will help to correctly record the data when articles assess multiple intervention or comparators, or more than one taxonomic group or biodiversity metric. Including multiple observations when they are provided in a single article will allow us to use all available data and thus estimate more precise effect sizes than using only a single pair of observations from each article [40]. We provide clear explanations of how we are going to record and code the extracted data in Appendix C. One reviewer will enter the data and a second reviewer will validate the accuracy and completeness of the data entry, for at least 80% of entries. If there is any disagreement between reviewers, the review team will discuss these to find agreement and make modifications to the database as necessary.

The extracted data will be used to assess the primary and secondary questions established. We will extract qualitative data on bibliography information (e.g., authors, publication year, title); Study_ID (i.e., numeric ID associated with each unique article, assigned during the screening process); Control-Intervention ID (i.e., numeric ID associated with each unique intervention or comparator, assigned during the coding process); dominant crop common name (e.g., maize, rice, banana); Experiment ID (i.e., numeric ID associated with each intervention-comparator comparison group, assigned during the extraction process to identify e.g., matching sampling dates in a repeat sampling design, matching study location); crop type (common name, scientific name, and Food and Agricultural Organisation of the United Nations commodity group); agricultural system as intervention or comparator (e.g., intercropping, monoculture, agroforestry, crop rotation, set aside); common and scientific name of the taxa sampled (e.g., ants, Formicidae); functional group sampled if specified (e.g., pest, decomposers; predator); biodiversity outcome metric (e.g., species richness, abundance, Shannon’s diversity index); the sampling method (e.g., transect, trap), pesticide use (yes or no, and kg/ha), fertilizer use (yes or no), fertilizer chemical (yes or no), soil management (e.g., tillage, no tillage, slash and burn); landscape characteristics (e.g., % agricultural land use, climate); and location of the study (i.e., country/state and geographical coordinates).

The quantitative outcome data that will be recorded include: biodiversity outcome means; biodiversity outcome variance measure (e.g., standard deviation, standard error of the mean, interquartile range, confidence intervals); number of samples collected; crop yield mean (e.g., kg/ha, g/plant), crop yield variance (e.g., standard deviation, standard error of the mean); farm size (in hectares); length of time that the land has been in current state (in years); and duration of the sampling period (in days, from start to finish). Data on biodiversity outcomes and crop yield will be extracted directly from publication text, tables, figures, or supplementary information. Data from figures will be extracted using GetData Graph Digitizer 2.26 or WebPlotDigitizer v4.2. Where data values or units are unclear or not provided (e.g., the meaning of the error bars in figures), the authors of the corresponding article will be contacted by email. If the authors do not respond, the data entry will be removed.

We will extract the data from all interventions and comparators if an article reports outcomes for multiple interventions (e.g., agroforestry and intercropping), or multiple comparator systems (e.g., simplified agricultural systems and natural). When outcomes are presented for multiple years (and no average across years is provided), we will include only the data of the last year assessed; otherwise, we will include the average across years. When outcomes are presented for multiple crop stages (e.g., flowering and non-flowering) or survey days (e.g., 25 and 55 days after sown) for the same year, and the average from across the study is not provided, we will extract all of the outcomes separately. When outcomes are reported at different distances away from the sampling plot, we will only include the data collected closest to the sampling plot. When biodiversity outcomes are reported for multiple taxa groups (e.g., ants, birds, and bees), functional groups (e.g., pests and decomposers), sample methods (e.g., vacuums and pitfall traps), and/or locations, we will enter each one separately in the database. When studies report outcomes for different life stages (e.g., adult, larvae, pupae), we will record only the most advanced stage. In the case where outcomes are disaggregated by functional group, and further disaggregated by taxonomic group, we will record the outcomes by functional group only. When a study presents multiple geographical coordinates for the same intervention, comparator, taxa, functional group or biodiversity measure, and these points are in the same region, we will record geographic coordinates of the centroid only. If a study does not present the exact location of the study area (i.e., in geographical coordinates), we will use the description of the study location to identify the proximate geographic coordinates, making sure to locate the points in agricultural or natural areas as described in each article.

After the data extraction process, we will reorganize the database using an R script so that each row of data contains observations from one comparator and an associated intervention from the same article. This procedure will facilitate the calculation of effect sizes.

## 8. Step 5: Observations Validity Assessment

Each observation (i.e., comparison between outcomes from one comparator and an associated intervention) of all included articles will be assessed to determine its internal validity (i.e., the probability to present bias). We will not consider external validity (i.e., how generalizable the observation is) as this is likely to vary geographically and with the population and intervention/comparator assessed.

Table 3 includes the list of criteria for the observation validity assessment. These criteria represent what we will consider relevant to rate the quality of the included observations, based on objective measures of internal validity. Sources of potential bias accounted or corrected for through meta-analytical procedures are excluded from Table 3 (e.g., different sample sizes between interventions and comparators; non-independence of effect sizes within studies).

Each observation will be scored as having a “Low”, “High”, or “Unclear” risk of bias relating to each of the criterion in Table 3. Observations that present “High risk” and/or “Unclear” for two or more assessment criteria will be classified as having a high risk of bias. All the necessary data to score each observation against the validity criteria will be recorded during the data extraction process. The quality and consistency of the recorded information will be checked as described in the data extraction and coding section.

## 9. Step 6: Identification of Potential Effect Modifiers/Reasons for Heterogeneity

The review team will extract the potential effect modifiers directly from the included studies or will re-classify the extracted data to generate the information. The following list details the factors (moderators) that will be considered as potentially causing effect size variation between and within the included studies:

- Crop type
- Population taxonomic group
- Functional group
- Type of intervention practice (i.e., type of diversified system)
- Biodiversity outcome metric
- Fertilizer application
- Pesticide application
- Soil management
- Landscape context (i.e., composition, structure)

Additional effect modifiers may be included to this list as the review proceeds. The list of potential effect modifiers was compiled based on evidence reported in the literature including in previous related meta-analysis [26,37,38].

## 10. Step 7: Data Synthesis and Presentation

Results of the data analysis will synthesize evidence of farm diversity, pesticide and fertiliser management effect on a variety of taxa, functional groups, and across biodiversity metrics. Additionally, the analysis will also quantify the effect of different farm diversification strategies on agricultural yields. A narrative synthesis will be performed in order to summarize, describe and present the results. A descriptive statistic using figures and tables will be conducted to visualize the distribution of the data across countries, taxa groups, functional groups, intervention practices, crop types and quality of observations (assessed against our internal validity criteria).

We will use effect sizes based on means to conduct a quantitative meta-analysis of the impact of interventions and comparators on biodiversity outcomes and yield. We will calculate the effect sizes using: (i) simplified farming systems as the control and diversified farming systems as the intervention, or (ii) natural habitat as the control and diversified farming systems as the intervention. Then, we will conduct the meta-analyses in R-4.0.0 [42] using the package *metafor* [43]. We will perform random-effect meta-analysis, to account for between-study and within-study variance [44]. Effect sizes will be pooled using multi-level mixed effects models to obtain overall mean effect sizes. Meta-regression procedures will be performed to examine the moderator effect of potential modifiers on biodiversity outcomes. The mean effect of intervention/control systems and the variance values from the meta-analysis will be shown using forest plots.

The presence of publication bias will be identified and assessed using visualization and statistical methods proposed by Nakagawa and Santos [45] for biological meta-analyses. We will use Cook’s distance or hat values and other established methods to identify potential influential cases and extreme observations [46]. We will conduct sensitivity analysis to compare the results from the meta-analytical models fitted with all the included studies and the models fitted after excluding outliers. All data and codes used in the analysis will be made available on publication of the results.

The compiled database of effect sizes will be made publicly available in raw format and through an interactive and user-friendly website which provides a comprehensive overview of the effects of diversified farming systems on terrestrial biodiversity outcomes and yield. We will also provide the literature included in the analysis for transparency. This would assist researchers and policymakers to assess the effects of different strategies and design improved interventions for their country, crop, farming system, or taxa of interest.

## Figures and Tables

**Table 1:** Search strings that will be used to identify relevant articles in two global repositories. The wildcard “*” is used to represent unknown characters or no character, and “?” to represent a single character or no character, to allow for variable spellings and truncations of search terms.

Repository	Search String
Scopus	TITLE-ABS-KEY (“agricultur*” AND “biodiversity”) AND TITLE-ABS-KEY (“agro?ecology” OR “agro?biodivers*” OR “agroforestry” OR “border plant*” OR “riparian buffer” OR “woodlot” OR “hedgerow” OR “cover crop*” OR “crop rotation” OR “crop divers*” OR “inter?crop*” OR “mixed crop*” OR “cultivar mixture” OR “plant divers*” OR “polyculture” OR “tree divers*” OR “variet* diversity” OR “fallow” OR “field margin*” OR “grass strip*” OR “*flower strip*” OR “insect* strip” OR “conservation strip” OR “vegetation strip” OR “catch crop” OR “inter?crop*” OR “crop variety” OR “crop sequenc*” OR “mixed farming” OR “land sparing” OR “landscape heterogeneity” OR “heterogeneous landscape” OR “landscape diversi*” OR “divers* landscape” OR “homogeneous landscape” OR “landscape homogeneity” OR “landscape complexity” OR “simplif* landscape” OR “complex landscape” OR “multi?function* landscape” OR “integrated crop-livestock” OR “integrated crop-forest” OR “land sharing”) AND TITLE-ABS-KEY (“ richness” OR “ abundance” OR “species diversity” OR “functional diversity” OR “index”) AND TITLE-ABS-KEY (“crop yield” OR “crop production”) AND (LIMIT-TO (LANGUAGE, “English”)).
Web of Science	Web of Science search string: (TS= (“agricultur*” AND “biodiversity”) AND TS= (“agro?ecology” OR “agro?biodivers*” OR “agroforestry” OR “border plant*” OR “riparian buffer” OR “woodlot” OR “hedgerow” OR “cover crop*” OR “crop rotation” OR “crop divers*” OR “inter?crop*” OR “mixed crop*” OR “cultivar mixture” OR “plant divers*” OR “polyculture” OR “tree divers*” OR “variet* diversity” OR “fallow” OR “field margin*” OR “grass strip*” OR “*flower strip*” OR “insect* strip” OR “conservation strip” OR “vegetation strip” OR “catch crop” OR “inter?crop*”OR “crop variety” OR “crop sequenc*” OR “mixed farming” OR “land sparing” OR “landscape heterogeneity” OR “heterogeneous landscape” OR “landscape diversi*” OR “divers* landscape” OR “homogeneous landscape” OR “landscape homogeneity” OR “landscape complexity” OR “simplif* landscape” OR “complex landscape” OR “multi?function* landscape” OR “integrated crop-livestock” OR “integrated crop-forest” OR “land sharing”) AND TS= (“richness” OR “abundance” OR “species diversity” OR “functional diversity” OR “index”) AND TS= (“crop yield” OR “crop production”)) AND LANGUAGE: (English)

**Table 2:** Control and intervention systems descriptions.

System	Description	Type
Agroforestry	Following Beillouin et al. [27], agroforestry satisfies three conditions: (i) at least two plant species interact biologically, (ii) at least one of the plant species is a woody perennial, and (iii) at least one of the plant species is managed for forage, annual or perennial crop production. Includes alley cropping with trees, shade monoculture, silvo-pasture.	Intervention
Cover crops	Following Beillouin et al. [27], plant grown in addition to the main crop for agronomic purposes, e.g., to manage soil erosion, pests, soil fertility or soil quality. The associated plant could be harvested or not, perennial or not.	Intervention
Crop rotation	Following Beillouin et al. [27], recurrent succession of a set of selected crops grown on a particular agricultural land each season or each year according to a definite plan.	Intervention
Diversified other	Diversity-based practices not included in other categories. Includes combinations of single practices, such as crop rotation and cover crops used in unison, and integrated crop-livestock systems.	Intervention
Embedded natural	Land on-farm not used for farming and where non-crop plants are sown or regenerated naturally to benefit biodiversity or for other environmental purposes. Includes fallow (regular, >6 months), field margins, hedgerows, riparian buffers, set aside, vegetation strips, flower strips.	Intervention
Intercropping	Adapted from Beillouin et al. [27], the simultaneous cultivation in the same field of two or more crop species, varieties, or cultivars, for all or part of their growth cycle. All crops are harvested.	Intervention
Monoculture	The cultivation of a single crop species or variety in the same plot at the same time or continually in different seasons.	Control
Simplified other	Relatively low diversity (usually only 2 species) agroforestry, cover crop, crop rotation or intercropping, for studies comparing these against the same cropping system planted with relatively high diversity (usually 3 or more species). Also used for cropped areas with no embedded natural features (e.g., hedgerows, vegetation strips) when compared against cropped areas with these embedded natural natures.	Control
Abandoned farmland	Abandoned cropland left to rewild.	Control
Natural	Forests, shrubland or grassland that is not commercially harvested and which, if managed, is managed for conservation purposes. Can include primary or secondary vegetation growth. Includes fynbos, natural or semi-natural grassland, remnant vegetation, secondary successional habitat.	Control

**Table 3:** List of criteria for observation validity assessment.

Assessment Criteria	Low Risk of Bias	High Risk of Bias	Unclear Risk of Bias	Type of Bias Addressed
1. Intervention and comparator sample size	Total sample size >= 5	Total sample size < 5	-	Selection bias
2. Are the interventions and comparators matching at the same site (i.e., same climate conditions, weather events, soil type)	Yes	No	Insufficient data to permit assessment of ‘Low risk’ or ‘High risk’ bias	Selection bias
3. Time the interventions and comparators plots have been in this state	>=1 year	<1 year	Insufficient data to permit assessment of ‘Low risk’ or ‘High risk’ bias	Selection bias
4. Can the intervention clearly be classified?	Yes. The intervention can be classified as one of the diversified systems specified in Table 2	No. The intervention was described by the article as “polyculture”.	-	Selection reporting, performance bias,

## Data Availability

No new data were created or analyzed in this study. Data sharing is not applicable to this article.

## Appendix A

**Table A1:** ROSES form for systematic review protocols. Version 1.0 (Haddaway et al., 2017).

Section/Sub-Section	Topic	Description	Further Explanation	Checklist/ Meta-Data	Author Response
Title	Title	The title must indicate that it is a systematic review protocol, and must indicate if it is an update/amendment: e.g., “A systematic review update protocol...”.	The title should normally be the same or very similar to the review question.	Meta-data	The impact of diversified farming practices on terrestrial biodiversity outcomes and agricultural yield worldwide: a systematic review protocol
Type of review	Type of review	Select one of the following types of review: systematic review, systematic review update, systematic review amendment, systematic review from a systematic map.	See Collaboration for Environmental Evidence (CEE) Guidance on amendments and updates.	Meta-data	Systematic review
Authors contacts	Authors contacts	The full names, institutional addresses, and email addresses for all authors must be provided.		Checklist	Yes
Abstract	Structured summary	Abstract must include the context and purpose of the review, including the review question; methods, how the review will be conducted and the outputs that are expected (specifically mention search strategy, inclusion criteria, critical appraisal, data extraction and synthesis).		Checklist	Yes
Background	Background	Describe the rationale for the review in the context of what is already known. Protocol must indicate why this study was necessary and what it aims to contribute to the field.	A theory of change and/or conceptual model can be presented that links the intervention or exposure to the outcome.	Checklist	Yes
Stakeholder engagement	Stakeholder engagement	The planned/actual role of stakeholders throughout the review process (e.g., in the formulation of the question) must be described and explained (using a broad definition of ‘stakeholder’, including e.g., researchers, funders and other decision-makers)		Checklist	Yes
Objective of the review	Objective	Describe the primary question and secondary questions (when applicable).	The primary question is the main question of the review. Secondary questions are usually linked to sources of heterogeneity (effect modifiers).	Checklist	Yes
	Definitions of the question components	Break down and summarise question key elements e.g., population, intervention(s)/exposure(s), comparator(s), and outcome(s).		Meta-data	Population: Terrestrial biodiversity in agricultural fields worldwide such as Bacteria, Animals, Fungi, Plants, etc. Intervention: Diversified farming practices at temporal and/or spatial scales (e.g., agroforestry, crop rotation, intercropping) Comparator: Simplified farming practices (e.g., monoculture), and/or natural habitats (e.g., natural grasslands, primary or secondary forest) Outcomes: Biodiversity outcome metrics (e.g., Abundance, Species Richness, Shannon’s diversity index, Percentage Fungi Colonisation, etc.) and Agricultural yield (i.e., crop yield in kg/ha).
Methods					
Searches	Search strategy	Detail the planned search strategy to be used, including: database names accessed, institutional subscriptions (or date ranges subscribed for each database), search options (e.g., ‘topic words’ or ‘full text’ search facility), efforts to source grey literature, other sources of evidence (e.g., hand searching, calls for evidence/submission of evidence by stakeholders).	Details regarding search strategy testing should be provided.	Checklist	Yes
	Search string	Provide Boolean-style full search string and state the platform for which the string is formatted (e.g., Web of Science format).		Meta-data	See Table 1
	Languages—bibliographic databases	List languages to be used in bibliographic database searches.		Meta-data	English
	Languages—grey literature	List languages to be used in organizational websites searches and web-based search engines.		Meta-data	n/a
	Bibliographic databases	Provide the number of bibliographic databases to be searched.		Meta-data	2
	Web—based search engines	Provide the number of web—based search engines to be searched.		Meta-data	0
	Organisational websites	Provide the number of organisational websites to be searched.		Meta-data	0
	Estimating the comprehensiveness of the search	Describe the process by which the comprehensiveness of the search strategy was assessed (i.e., list of benchmark articles).		Checklist	Yes
	Search update	Describe any plans to update the searches during the conduct of the review.	Optional. A search update is good practice if original searches were performed more than two years prior to review completion.	Checklist	Yes
Article screening and study inclusion criteria	Screening strategy	Describe the methodology for screening articles/studies for relevance/eligibility.		Checklist	Yes
	Consistency checking	Describe clearly the process for checking consistency of decisions including the levels at which consistency checking will be undertaken and estimated proportion of articles/studies that will be screened and checked for consistency by two or more reviewers (e.g., Titles (10%), abstracts (10%), full text (10%)).		Checklist	Yes
	Inclusion criteria	Describe the inclusion criteria used to assess relevance of identified articles/studies. These must be broken down into the question key elements (e.g., relevant subject(s), intervention(s)/exposure(s), comparator(s), outcomes, study design(s)) and any other restrictions (e.g., date ranges or languages).		Checklist	Yes
	Reasons for exclusion	State that you will provide a list of articles excluded at full text with reasons for exclusion.		Checklist	Yes
Critical appraisal	Critical appraisal strategy	Describe here the method you propose for critical appraisal of study validity (including assessment of individual studies and the evidence base as a whole).		Checklist	Yes
	Critical appraisal used in synthesis	Describe how the information from critical appraisal will be used in synthesis.		Checklist	Yes
	Consistency checking	Describe how repeatability of critical appraisal of study validity will be tested.		Checklist	Yes
Data extraction	Meta-data extraction and coding strategy	Describe the method for meta-data extraction and coding for studies (potentially providing forms/data sheets (ideally piloted), list if variables to be extracted as meta-data and those that will be coded).		Checklist	Yes
	Data extraction strategy	Describe the method for extraction of qualitative and/or quantitative study findings (potentially providing forms/data sheets (ideally piloted)).		Checklist	Yes
	Approaches to missing data	Describe any processes for obtaining and confirming missing or unclear information or data from authors.		Checklist	Yes
	Consistency checking	Describe how repeatability of the meta-data/data extraction process will be tested.		Checklist	Yes
Potential effect modifiers/reasons for heterogeneity	Potential effect modifiers/reasons for heterogeneity	Provide a list of and justification for the effect modifiers /reasons for heterogeneity that will be considered in the review. Also provide details of how the list was compiled (including consultation of external experts).	The list should not be exhaustive but a short list of those variables thought to be most important and amenable to analysis.	Checklist	Yes
Data synthesis and presentation	Type of synthesis	State the type of synthesis conducted as part of the systematic review (narrative only, narrative and quantitative, narrative and qualitative, narrative, qualitative and quantitative, narrative and mixed-methods).		Meta-data	Narrative and quantitative
	Narrative synthesis strategy	Describe methods to be used for narratively synthesising the evidence base in the form of descriptive statistics, tables (including any map databases) and figures.	Vote-counting (tallying of studies based on the direction or significance of their findings) must be avoided. Must include a summary of the outputs of critical appraisal of the evidence base as a whole.	Checklist	Yes
	Quantitative synthesis strategy	If data are appropriate for quantitative synthesis, describe planned methods for calculating effect sizes, methods for handling complex data, statistical methods for combining data from individual studies, and any planned exploration of heterogeneity (e.g., sensitivity analysis, subgroup analysis and meta-regression). If all studies may not be selected for synthesis explain criteria for selection (e.g., incomplete or missing information).	Compulsory if appropriate for data	Checklist	Yes
	Qualitative synthesis strategy	Describe methods to be used for synthesising qualitative data and justify your methodological choice. Describe if and how you plan to analyse subgroups/subsets of data. If all studies may not be selected for synthesis explain criteria for selection (e.g., incomplete or missing information).	Compulsory if appropriate for data	Checklist	Yes
	Other synthesis strategies	Describe any other approaches to be used for synthesising data or combining qualitative and quantitative synthesis (e.g., mixed-methods) and justify your methodological choice.	Compulsory if appropriate for data	Checklist	N/A
	Assessment of risk of publication bias	Describe planned methods for examining the possible influence of publication bias on the synthesis.	For quantitative syntheses this may be done using diagnostic plots or statistical tests	Checklist	Yes
	Knowledge gap identification strategy	Describe the methods to be used to identify and/or prioritise key knowledge gaps (unrepresented or underrepresented subtopics that warrant further primary research).	Optional	Checklist	No
	Demonstrating procedural independence	Describe the role of systematic reviewers (who have also authored articles to be considered within the review) in decisions regarding inclusion or critical appraisal of their own work.	Reviewers who have authored articles to be considered within the review should be prevented from unduly influencing inclusion decisions, for example by delegating tasks appropriately.	Checklist	N/A
Declarations	Competing interests	Describe any financial or non-financial competing interests that the review authors may have.		Checklist	N/A

## Appendix B. Search String Development Process

### Search Strings Trialled during the Methodology Development Process

We trialled several alternative search strings with the aim of ensuring the search retrieved all relevant articles while reducing the number of non-relevant articles picked up. Search strings trialled, formatted for Scopus, include:

- String 1: TITLE-ABS-KEY ((agriculture AND biodiversity AND (Agrobiodiversity OR Agro-biodiversity OR Agroforestry OR Cover crop OR Crop diversity OR Diversification OR Ecological diversity OR Fish diversity OR Intercropping OR Land use and cover diversity OR Landscape complexity OR Livestock diversity OR Mixed cropping OR Mixed farming systems OR Multifunctional agriculture OR Multifunctional farm OR Multifunctional landscape OR Pollinator diversity OR Pollinator richness OR Polyculture OR Seed diversity OR Set-aside OR Soil biodiversity OR Varietal diversity) AND (biodiversity loss OR biodiversity erosion OR species extinction OR species loss OR habitat loss OR decrease in biodiversity OR wild biodiversity OR increase in biodiversity OR biodiversity conservation OR loss of biodiversity OR conservation of biodiversity OR erosion of biodiversity loss of species OR loss of habitat OR conservation of habitat OR conservation of species OR change in biodiversity OR species conservation OR habitat conservation))).
- String 2: TITLE-ABS-KEY (“agricultur*” AND “biodiversity”) AND TITLE-ABS-KEY (“agrobiodivers*” OR “agro-biodivers*” OR “agroforestry” OR “border plant*” OR “cover crop*” OR “crop rotation” OR “crop divers*” OR “fallow” OR “field margin*” OR “grass* strip*” OR “*flower strip*” OR “insectary strip” OR “intercrop*” OR “mixed crop*” OR “plant divers*” OR “polyculture” OR “tree divers*” OR “riparian buffer” OR “woodlot”) AND TITLE-ABS-KEY (“species richness” OR “species abundance” OR “species diversity” OR “functional diversity” OR “index”) AND TITLE-ABS-KEY (“crop yield” OR “crop production”) AND (LIMIT-TO (LANGUAGE, “English”)).

In Scopus, String 1 retrieved 124 articles, most of which did not include a quantitative comparison of diversified farming compared to simplified farming or natural systems.

String 2 retrieved 1303 articles, and a screening of the first ~100 indicated most contained a quantitative comparison of a suitable intervention and comparator, but did not report effects on crop production or yield. Therefore, we adapted the search string to constrain it to articles that also contained ‘crop production’ or ‘crop yield’ in the title, abstract or keywords.

## Appendix C. Draft Extraction Sheet

**Table A2:** Extraction sheet. Includes the quantitative and qualitative meta-data to be extracted along, with a clear explanation of how to record and code the data for each column.

Data	Code	Description
Study_ID	ID	Take from the meta-analysis list of articles retrieved spreadsheet.
Comparison_ID	Comparison_ID	If a study reports outcomes for multiple intervention (e.g., agroforestry and intercropping), or comparator systems (e.g., simplified agricultural systems and natural), data from all interventions and comparators will be extracted and coded separately. For example, for multiple controls, put C1, C2 … giving a unique number to each unique control. For the intervention practices, put T1, T2, …. using same logic. This will be used to correctly link comparators and intervention combinations within a single study, for analysis. Notes: If the taxa group, biodiversity measure and experiment stage are the same, then use the same C value and T values. Change C and T values if other parameters like location, pesticide use, etc. change.
Crop type (main species common to all treatments—common name) OR natural land type	Crop	For cropped land, put main crop species common to all treatments. Use COMMON name. For non-cropped land, put vegetation type e.g., grassland, forest, hedgerow.
Crop type (main species common to all treatments—scientific name)	Crop_scientific	For cropped land, put main crop species common to all intervention systems. Use SCIENTIFIC name.
All crops common name (separate with comma)	crops_all_common	List all crops in the intervention systems by common name, with crops separated by a comma.
All crops scientific name (separate with comma)	crops_all_scientific	List all crops in the intervention systems by scientific name, with crops separated by a comma.
Crop scientific name taxonomic level	crops_all_scientific_level	List the taxonomic level of classification separated by a coma. E.g., Species OR Genus; Species OR Genus.
Wild taxa common name (separate with comma)	taxa_common	List all (wild) species assessed by common name, with names separated by a comma.
Wild’ taxa assessed scientific name at SPECIES level ideally (separate with comma)	taxa_scientific	List all (wild) species assessed as per column by scientific name, separated by a comma.
Wild taxa taxonomic level	taxa_scientific_level	List the taxonomic level of classification separated by a coma. E.g., Species OR Genus; Species OR Genus.
Cropping system	System_raw	Find match within definitions of terms in drop down list on the KEY tab (a tab providing lists of terms and their definition to help standardise data entry). Use authors’ classification if there’s no perfect fit into one of the options on the dropdown list (and then add to the ‘KEY’ tab any new terms).
Cropping system OR natural land description	System_details	Provide the name of the treatment as written in the paper, including abbreviation, and (for cropland) a list of crop and tree species cropped in combination with main crops. Include also an explanation of the cropping or natural system, if helpful for understanding context.
Crop species richness	Crop_species_richness	Number of crop species in the cropping system.
Crop variety richness	Crop_variety_richness	Number of crop varieties in the cropping system (minimum).
Management system	Management_system_raw	Summary term to describe agricultural inputs.
Taxa assessed	Taxa	Common or scientific name, order, family, genus of the organisms sampled.
Taxa Details	Taxa_details	Life stage, scientific name or any other important detail.
Taxa class	Taxa_Class	Taxonomic Class of the assessed biodiversity.
Functional group	Functional_group	Functional group classification considering the information provided by the study. See definitions in the “KEY”.
Crop name standardised for analysis	Crop_clean	Automatic classification see “Crop name standardised for analysis” column in “KEY”.
Cropping system reclassification for analysis	System	Automatic classification see “Reclassification for analysis (detailed—analysis of monoculture versus other)” column in “KEY”.
Management system reclassification for analysis	Management_system	Automatic classification see “Management system reclassification for analysis” column in “KEY”.
Comparison class for analysis	Comparison_class	Specifies whether it is simplified agriculture (i.e., monocrop or similar), diversified agriculture, or natural land (grassland, forest, marsh), for use in data analysis stage. Automatic classification, see “Reclassification for analysis (detailed—analysis of monoculture versus other)” column in “KEY”.
Simplified taxa for analysis	Taxa_group	Automatic classification, see “Simplified taxa for analysis” column in “KEY”.
Biodiversity measure	B_measure	Biodiversity outcome. See options in “KEY”.
Biodiversity measure #	B_value	Use mean values. If only median is given, enter data setting “median, IQR” in column B_error_measure.
Biodiversity variance measure	B_error_measure	Specify the error measure (Median, Interquartile range, standard error, standard deviation, confidence intervals).
Biodiversity variance #	B_error_value	Median and/or other variance values.
Biodiversity variance IQR lower OR CI lower	B_error_range_l	Put lower Interquartile range or Confidence interval.
Biodiversity variance IQR upper or CI upper	B_error_range_u	Put upper Interquartile range or Confidence interval.
Biodiversity SD	B_SD	Standard deviation value calculated using the formulas describe in “KEY”.
Relation to ground	B_ground	See options in “KEY”.
Yield	Yield	Yield or mean yield corresponding to the season(s) for which biodiversity outcome data are recorded.
Yield metric	Yield_metric	Kg/ha where possible, otherwise insert the metric used, e.g., g/plant.
Yield SD	Yield_SD	Record standard deviation in yield, where provided. If standard errors or other variance values are provided, convert these to standard deviation before entry.
Farm size (ha)	Farm_size	
Farming context	Farm_context	Any important detail e.g., experimental, commercial.
Fertiliser use	Fertiliser	yes/no.
Chemical fertiliser use	Fertiliser_chem	yes/no.
Pesticide / herbicide use	Pesticide	yes/no.
Pesticide quantity (kg/ha)	Pesticide_quantity	
Soil management	Soil_management	See options in “KEY”.
Time land has been in this state (years, minimum)	Time_state	
Length of study (days)	Study_length	
Landscape Context	Landscape_context	Any important detail (e.g., % agricultural land use).
Sampling unit	Sampling_unit	Sampling details (e.g., leaf, 10x10m quadrant, field).
Number of samples	N_samples	Number of distinct sampling units where biodiversity was sampled, e.g., ants on 4 leaves on 5 trees at each of 10 fields means sample size = 4 × 5 × 10 = 200.
Location	Location	Place, City.
Country	Country	
Lat	Lat	If there is multiple Lat/Longs for the same intervention, comparator, taxa, functional group or biodiversity measure, and these points are in the same region, then we will take the middle point.
Long	Long	If there is multiple Lat/Longs for the same intervention, comparator, taxa, functional group or biodiversity measure, and these points are in the same region, then we will take the middle point.
Notes	Notes	“Raw Data”: copy pasted values from paper “Estimated”: calculated from graph Put which table/ figure the data is taken from.
Stage of measurement	Experiment_stage	Use to record a unique ID for each comparator-intervention pairing that needs to be maintained in analysis. For example: when biodiversity outcomes are reported for multiple taxa groups (e.g., ants, birds, and bees), functional groups (e.g., pests and decomposers), sample methods (e.g., vacuums and pitfall traps), and/or locations, we will enter each one separately in the database, and code with the same number the intervention and comparator outcomes of the same taxa group (e.g., intervention ants = code: 1; comparator ants = code: 1; intervention birds = code: 2; comparator birds = code:2; intervention bees = code: 3; comparator bees = code:3), functional group (e.g., intervention pest = code: 1; comparator pest = code: 1; intervention decomposers = code: 2; comparator decomposers = code: 2), sample methods (e.g., intervention vacuums = code: 1; comparator vacuums = code: 1; intervention pitfall traps = code: 2; comparator pitfall traps = code: 2); and/or location (e.g., intervention in location A = code: 1; comparator in location A = code: 1; intervention in location B = code: 2; comparator in location A = code: 1).
Data entry person	Data_entry	Reviewer who recorded the data.
Data validation person	Data_validation	Reviewer who checked the data.
Ecological process?	Process	Usually taken from abstract and/or discussion that summarizes main results & ecological explanation behind.
Crop FAO group	Crop_FAO	
Crop duration type	Crop_ann_pen	annual/perennial.
Crop woodiness	Crop_woodiness	
simplified location	Location_simplified	
